# An immunotherapeutic artificial vitreous body hydrogel to control choroidal melanoma and preserve vision after vitrectomy

**DOI:** 10.1126/sciadv.adh1582

**Published:** 2023-11-01

**Authors:** Muchao Chen, Jiaying Hu, Huiqin Gao, Jingjing Shen, Ting Wei, Jing Yao, Yan Zhang, Ping Gu, Zhuang Liu, Qian Chen

**Affiliations:** ^1^ Institute of Functional Nano and Soft Materials (FUNSOM), Jiangsu Key Laboratory for Carbon Based Functional Materials and Devices, Soochow University, 199 Ren’ai Road, Suzhou, Jiangsu 215123, P.R. China.; ^2^ Department of Ophthalmology, Ninth People's Hospital, Shanghai JiaoTong University School of Medicine, Shanghai 200011, P.R. China.; ^3^ Shanghai Key Laboratory of Orbital Diseases and Ocular Oncology, Shanghai 200011, P.R. China.; ^4^ Department of Ophthalmology and Vision Science, Eye & ENT Hospital, Shanghai Medical School, Fudan University, 83 Fenyang Road, Shanghai 200031, China.; ^5^ Department of Orthopedics, Shanghai Yangpu Hospital Affiliated to Tongji University, Shanghai 200090, China.

## Abstract

Choroidal melanoma, a common intraocular malignant tumor, relies on local radiotherapy and enucleation for treatment. However, cancer recurrence and visual impairment remain important challenges. Here, a therapeutic artificial vitreous body (AVB) hydrogel based on tetra-armed poly(ethylene glycol) was developed to control the recurrence of choroidal melanoma and preserve vision after vitrectomy. AVB loaded with melphalan (Mel) and anti–programmed cell death ligand-1 (αPDL1), was injected after surgical resection in the choroidal melanoma mouse model. Afterwards, the sequentially released Mel and αPDL1 from AVB could achieve a synergistic antitumor effect to inhibit tumor recurrence. AVB with similar physical properties to native vitreous body could maintain the normal structure and visual function of eye after vitrectomy, which has been evidenced by standard examinations of ophthalmology in the mouse model. Thus, the immunotherapeutic AVB may be a promising candidate as an infill biomaterial to assist surgical treatment of intraocular malignant tumors.

## Teaser

An immunotherapeutic artificial vitreous body was developed as a crucial surgical adjunct for choroidal melanoma treatment.

## INTRODUCTION

Choroidal melanoma is a kind of primary intraocular malignant tumor that occurs commonly in adults with a high lethality rate and poor therapeutic results (*1*–*3*). Commonly, plaque brachytherapy and proton beam radiotherapy have been used for local control of early choroidal melanoma in the clinic. However, these treatments still have many limitations. For example, plaque brachytherapy usually has uneven dose distribution, which is prone to create hot spots (high-dose areas) and cold spots (low-dose areas) (*4*, *5*), and has the risk to cause radiation fundus disease (*6*–*8*). Proton beam radiotherapy usually requires high cost to construct and maintain the proton center, which makes it impossible for patients to access the treatment (*9*). These radiations have limited efficacy in the treatment of larger choroidal melanoma (*10*, *11*). Besides, enucleation of the affected eyeball is a conventional treatment of larger choroidal melanoma, but it is a disfiguring procedure causing serious physical and mental consequences to patients (*12*, *13*). Different from enucleation, intraocular local surgical resections, especially vitrectomy as a high-precision microsurgical method to conserve as much vision as possible, has the potential to be used for eyeball preservation at the middle stage of choroidal melanoma (*14*–*16*). After surgery, expansile gases or silicone oil are usually used for medium- and long-term tamponades to facilitate the adhesion of detached neurosensory retina to the underlying retinal pigment epithelium (*17*, *18*). However, expansile gases for endotamponade usually cause severe inconvenience to patients, such as maintaining a prone posture in the special position for the long term (*19*). Silicone oil usually requires additional removal surgery at 3 to 6 months and is accompanied by potential complications, such as raised intraocular pressure (IOP), cataract formation, temporary loss of vision, and long-term retinal toxicity (*20*). Moreover, cancer cells often infiltrate the local lamellar sclera and easily remain at the edge of excised tissues, leading to a high recurrence rate after surgery (*21*, *22*).

Chemotherapy is often used after surgery to prevent the recurrence of tumors, but the therapeutic results are usually unsatisfactory (*23*, *24*). Recently, immunotherapy, as a revolutionary cancer treatment, has been shown to able to stimulate patients’ immune systems to attack cancer cells, providing a previously unidentified possibility for cancer treatment (*25*, *26*). Among them, immune checkpoint blockade using antibodies to block immune checkpoint pathways, such as the programmed cell death protein 1 pathway (PD-1/PD-L1), has been demonstrated to be an effective strategy in treating different types of cancers in the clinic (*27*). Considering the existence of complicated ocular structural barriers and low immunogenicity of the choroidal melanoma, intravenous administration of immune checkpoint antagonists is unable to achieve effective therapeutic benefit in previous researches (*28*), while intraocular injection of antagonists may cause many side effects, such as uveitis, endophthalmitis, and retinal detachment (*29*–*31*). Therefore, it is important to develop a biocompatible material that is able to serve as a vitreous substitute and drug reservoir simultaneously to preserve vision and sustainably release immunotherapeutic drugs to inhibit the recurrence of intraocular malignant tumors after surgical resection.

**Fig. 1.**
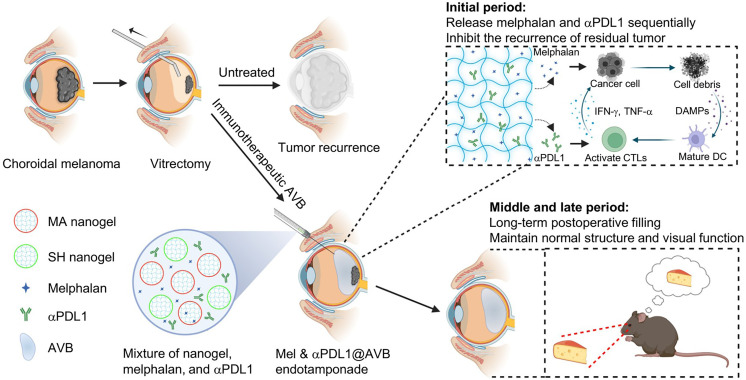
Schematic illustration of an immunotherapeutic AVB hydrogel for choroidal melanoma treatment after vitrectomy. An immunotherapeutic AVB hydrogel based on highly branched polymers of Tetra-PEG was developed to control the recurrence of choroidal melanoma and preserve vision after vitrectomy. Such AVB not only exhibited programmed release profiles of Mel and anti–programmed cell death ligand-1 (αPDL1) to synergistically inhibit the recurrence of residual tumors but also functioned as an ideal AVB to maintain the normal structure of the eye for visual function preservation after vitrectomy.

Herein, an artificial vitreous body (AVB) hydrogel based on highly branched polymers of tetra-armed poly(ethylene glycol) (Tetra-PEG) was developed to control the recurrence of choroidal melanoma and simultaneously preserve vision after vitrectomy (Fig. 1). Briefly, AVB, which functioned as a promising artificial vitreous body with extremely low swelling pressure, ultralow polymer content, and excellent biocompatibility, was prepared by mixing tetra-armed poly(ethylene glycol) with thiol termini (Tetra-PEG-SH) and maleimide termini (Tetra-PEG-MA). Such AVB could also act as a drug storage reservoir to sequentially release melphalan (Mel), a small-molecule drug used for arterial infusion chemotherapy against intraocular malignant tumors (*32*), and anti–programmed cell death ligand-1 (αPDL1), an immune checkpoint blockade antibody to restore T cell function by blocking the PD-1/PD-L1 pathway (*33*–*35*). The quickly released Mel could induce immunogenic cell death (ICD), which could further improve the immune response rate of αPDL1, achieving synergistic antitumor immune responses. In the mouse choroidal melanoma model, Mel&αPDL1@AVB effectively inhibited the postoperative tumor recurrence and prolonged the eyeball preservation of mice. Meanwhile, we demonstrated that AVB as the artificial vitreous body after vitrectomy could preserve vision functions of eyes as monitored via ophthalmotonometer slit-lamp, fundus fluorescein angiography (FFA), optical coherence tomography (OCT), and electroretinography (ERG) measure in the long term. Therefore, the immunotherapeutic AVB hydrogel should be a crucial surgical adjunct after vitreoretinal surgery for intraocular malignant tumors in the endophytic stage of eye (the stage that tumor growth has not yet caused irreversible damage to the eye).

## RESULTS

### Preparation and characterization of AVB

**Fig. 2.**
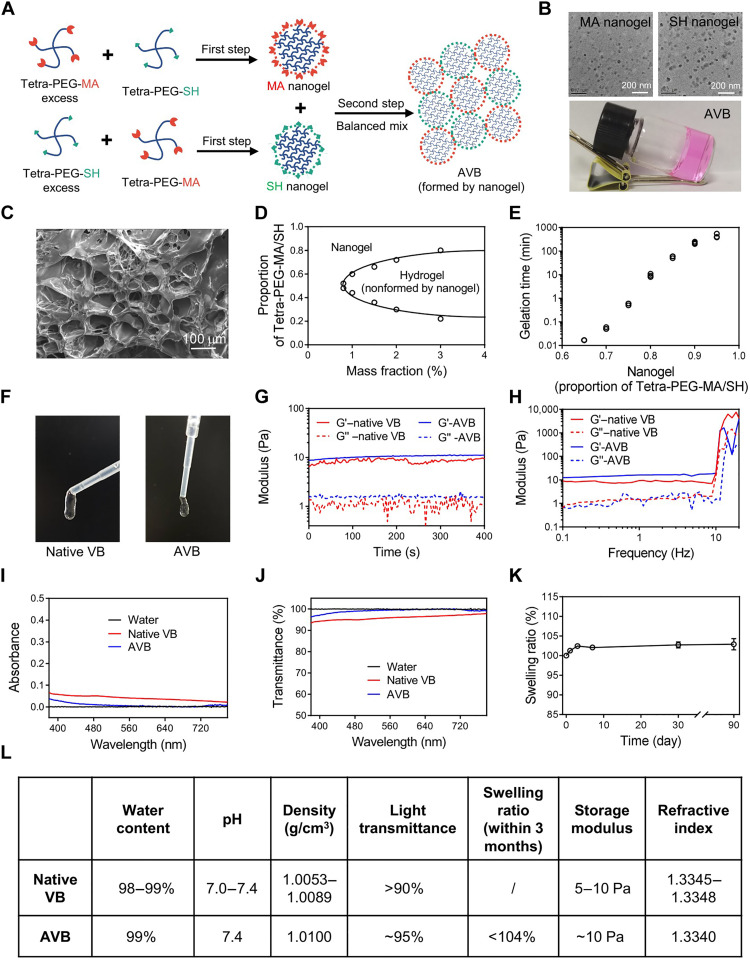
Preparation and characterization of AVB. (**A**) Scheme showing the preparation of AVB by a two-step process. First step: Excess Tetra-PEG-MA or Tetra-PEG-SH was blended with Tetra-PEG-SH or Tetra-PEG-MA to form MA nanogels or SH nanogels, respectively. Second step: The obtained MA nanogel and SH nanogel were mixed to form AVB. (**B**) Representative TEM images of the MA nanogel and SH nanogel. Photographs showing the AVB hydrogel (added with a colored dye for better observation). (**C**) Representative scanning electron microscopy image of the AVB hydrogel. (**D**) Nanogel-hydrogel phase diagram in the first step of mixing by changing the total mass fraction and the disproportionate commixture proportion of Tetra-PEG-MA and Tetra-PEG-SH. (**E**) Gelation time diagram in the second step after mixing an equal amount of MA nanogel and SH nanogel that were obtained with by different disproportionate proportion in the first step. Experiments were repeated three times. (**F**) Photographs of the native vitreous body (VB, donated by Department of Ophthalmology and Vision Science, Eye & ENT Hospital) and AVB. (**G** and **H**) Rheological behavior of native VB and AVB with time and oscillation frequency. G′, storage modulus; G″, loss modulus. (**I** and **J**) Absorbance curves and light transmittance of native VB and AVB in the range of visible light (380 to 780 nm). (**K**) The swelling ratio of AVB for 3 months. Experiments were repeated three times. Data are presented as means ± SEM (*n* = 3). (**L**) Table showing the physical properties of native VB and AVB.

AVB hydrogel was prepared by a two-step process (Fig. 2A). In the first step, excess Tetra-PEG-MA was blended with Tetra-PEG-SH at a proportion of 0.8/0.2 (total mass fraction = 1 wt %, proportion = [Tetra-PEG-MA]/[Tetra-PEG-SH]), forming maleimide groups surrounding the nanogel (MA nanogel) with a uniform hydrodynamic size at 38 nm (fig. S1). Similarly, another nanogel (SH nanogel) with a similar size covered with sulfhydryl groups was prepared by mixing Tetra-PEG-SH and Tetra-PEG-MA at a proportion of 0.8/0.2 (total mass fraction = 1 wt %, proportion = [Tetra-PEG-SH]/[Tetra-PEG-MA]). According to the ultraviolet-visible absorbance of Tetra-PEG-MA before and after being mixed with Tetra-PEG-SH and the concentration-dependent absorbance curve of Tetra-PEG-MA at 300 nm (fig. S2, A and B), we found that these two Tetra-PEG could react with each other completely in different proportions (fig. S3). Meanwhile, the obtained MA nanogel and SH nanogel were characterized by transmission electron microscope (TEM) imaging. As shown in Fig. 2B, the MA nanogel and SH nanogel exhibited similar spheriform morphology and uniform size (~30 nm). In the second step, the AVB hydrogel was synthesized by mixing an equal amount of MA nanogel and SH nanogel (Fig. 2B, colorless gel initially, with addition of colored dyes for better observation). As revealed by scanning electron microscopy imaging, the freeze-dried AVB hydrogel exhibited porous structure with pore sizes in the range of 10 to 100 μm (Fig. 2C).

Considering that the ratio of Tetra-PEG-MA and Tetra-PEG-SH and the total polymer concentration in the first step are the key variables during the preparation process of AVB hydrogel, we optimized the proportion of Tetra-PEG-MA/Tetra-PEG-SH and the total mass fraction in preparing the MA nanogel and SH nanogel. As shown in Fig. 2D, to form MA or SH nanogels, the [Tetra-PEG-MA]/[Tetra-PEG-SH] ratio should be larger than 0.6/0.4 or smaller than 0.4/0.6, respectively, otherwise rapid gelation of the whole solution would occur, in the first step of mixing. MA nanogel and SH nanogel, each of which was formed at the optimal [Tetra-PEG-MA]/[Tetra-PEG-SH] ratio of 0.8/0.2 and 0.2/0.8, respectively, exhibited an approximate gelation time at about 10 min upon mixing in the second step (Fig. 2E). On the other side, the storage modulus (G′) of AVB with different total mass fractions was measured (fig. S4). As the increase of total mass fraction, the G′ of AVB was also increased. Notably, the G′ of 1 wt % AVB was approximately 10 Pa, which is close to the G′ of the native vitreous body (*36*) (Fig. 2G). Therefore, a total mass polymer fraction of 1 wt % was chosen to conduct the final AVB.

To verify whether AVB would be suitable for the substitution of native vitreous body (VB) after vitrectomy, we comprehensively compared the physical properties of AVB and native VB. Both AVB and native VB appeared as transparent soft jelly (Fig. 2F) and exhibited similar rheological behaviors with the storage modulus stably kept at ~10 Pa (Fig. 2, G and H). In addition to the appearance and rheological behavior, the light transmittance of AVB is also important for filling after vitrectomy (*37*). As indicated in Fig. 2I, both AVB and native VB showed very low absorbance, with light transmittance of 90% for native VB and 95% for AVB in the range of visible light (380 to 780 nm) (Fig. 2J). In addition, during the swelling experiment, nearly no degradation of AVB was observed, and the swelling ratio of AVB remained below 104% for 3 months (Fig. 2K), demonstrating that AVB may not compress the eyeball during long-term tamponades. Moreover, the water content, pH value, density, and refractive index of AVB were further investigated. As summarized in Fig. 2L, both AVB and native VB showed similar water contents (avoiding adverse reactions such as elevated IOP caused by hydrogel swelling), pH values (permitting good biocompatibility), densities (ensuring no floating up in the eye), and refractive indexes (ensuring the normal image quality of the eyes) (*36*, *38*). In addition, AVB can achieve long-term storage using freeze-drying precursors, which would be convenience for future clinical use (fig. S5). Together, these results confirmed that AVB should be an excellent scaffold implant for long-term substitution of the native VB.

### Programmed release behavior of Mel and αPDL1 from the AVB

Although the polymer content of AVB is ultralow, it still has an abundant porous structure, allowing it excellent drug loading capacity. Mel and αPDL1 at therapeutic-relevant dosages were encapsulated into the AVB. Then, rhodamine B (RhoB)– and Cy5.5-labeled immunoglobulin G (IgG) (IgG-Cy5.5) as the substitution of Mel and αPDL1 were encapsulated into the AVB hydrogel, and drug-loaded AVB was sectioned and imaged by confocal imaging. As shown in fig. S6, strong signals from RhoB and Cy5.5 were observed, suggesting the successful encapsulation of both small and large molecules. Moreover, more than 95% of the Mel and αPDL1 could be loaded into the hydrogel tested by elution method (fig. S7). Then, RhoB- and IgG-Cy5.5–loaded AVB (RhoB&IgG-Cy5.5@AVB) was dispersed in phosphate-buffered saline (PBS). At different time points, AVB was collected, sectioned, and imaged by confocal imaging to visualize the release behaviors of encapsulated molecules (fig. S8). As expected, the fluorescence of RhoB was almost completely disappeared on the third day, and the fluorescence of IgG-Cy5.5 could still be detected on day 7, indicating that RhoB and IgG exhibited distinct release kinetics. Then, the quantitative release profiles of Mel and IgG from AVB were measured by high-performance liquid chromatography (HPLC) and enzyme-linked immunosorbent assay (ELISA), respectively. Approximately 74.5% of Mel was released within 1 day, while IgG exhibited more sustained release behavior, with 32.9% released in 1 day (fig. S9).

**Fig. 3.**
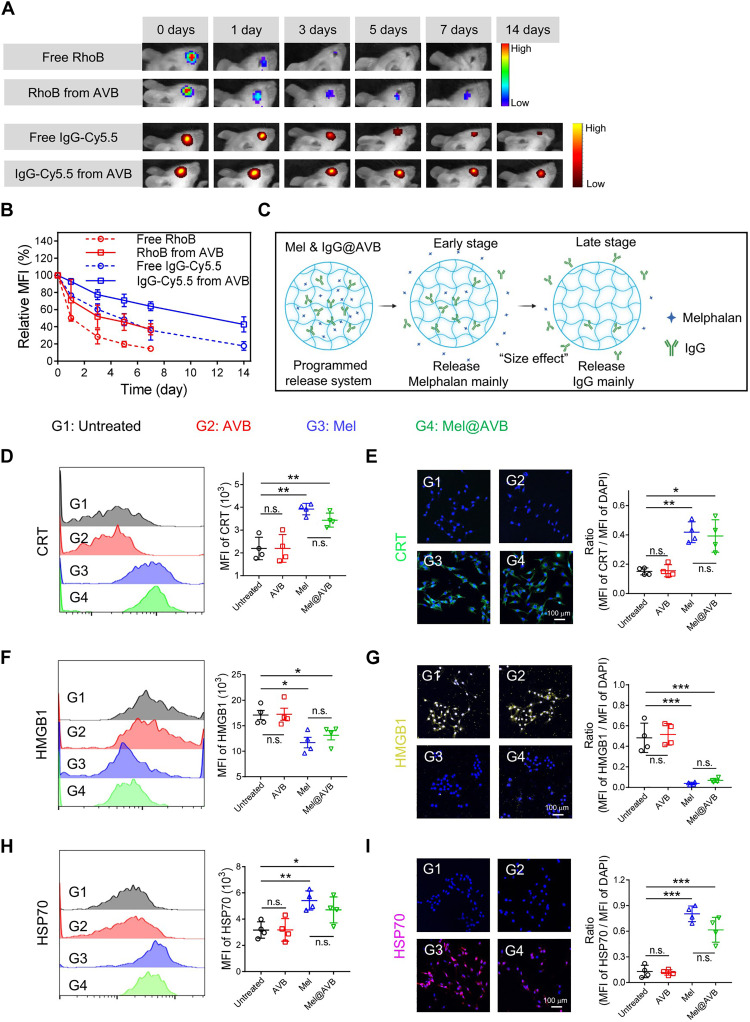
Programmed release behavior of Mel and αPDL1 from the AVB. (**A**) Fluorescence IVIS imaging depicting the retention of RhoB and IgG-Cy5.5 in the eye after injecting a mixture containing RhoB and IgG-Cy5.5 or AVB encapsulating RhoB and IgG-Cy5.5 at different time points. (**B**) Statistical fluorescence intensity of of RhoB and IgG-Cy5.5 retained in the eye. Data are presented as means ± SEM (*n* = 3). (**C**) Scheme showing the programmed release kinetics of loaded drugs from AVB. (**D**, **F**, and **H**) Representative fluorescence-activated cell sorting (FACS) histograms and statistical graphs of CRT, HMGB1, and HSP70 expression on B16F10 tumor cells with different treatments (G1: Untreated, G2: AVB, G3: Mel, and G4: Mel@AVB). Data are presented as means ± SEM (*n* = 4). MFI, mean fluorescence intensity. (**E**, **G**, and **I**) Immunofluorescence staining images and statistical graphs of CRT, HMGB1, and HSP70 expression on B16F10 tumor cells with different treatments. Data are presented as means ± SEM (*n* = 4). DAPI, 4′,6-diamidino-2-phenylindole.

Then, the distinct release kinetics of Mel and IgG were investigated in mice after intravitreal injection of free drug mixture or drug-loaded AVB by an in vivo fluorescence imaging system. Here, RhoB and IgG-Cy5.5 were still used as substitutes for Mel and αPDL1. As shown in Fig. 3 (A and B), for mice injected with the simple mixture, the fluorescence signals from RhoB and IgG-Cy5.5 were reduced to 14.4 and 35.9% of the initial signals, respectively, on day 7. In contrast, the fluorescence signals from RhoB and IgG-Cy5.5 in AVB-injected mice kept at 37.1 and 63.9% of the initial signals, respectively, on day 7. Thus, AVB while being able prolong the retention of drugs within eyes, could allow programmed release kinetics of loaded drugs depending on their molecular weights, with larger molecules released slower (Fig. 3C).

### Immunostimulatory efficacy induced by Mel@Gel

First, we investigated the inhibitory effect of Mel on both mouse melanoma cells B16F10 and human-derived choroidal melanoma cells MuM-2B. As shown in fig. S10, the proliferation of B16F10 and MuM-2B cells was obviously inhibited in the presence of Mel. Then, we investigated whether Mel could induce ICD, a distinct cell death pathway, during which a series of damage-associated molecular patterns (DAMPs) would be released, including the exposure of calreticulin (CRT) on the cell surface, the secretion of adenosine triphosphate (ATP) and high mobility group protein 1 (HMGB1), and the release of heat shock proteins (HSP70 and HSP90) (*39*, *40*). Specifically, B16F10 cells or MuM-2B cells were incubated with AVB, Mel, or Mel@AVB with a dosage of Mel at 80 μg/ml. Six hours later, the culture medium of B16F10 cells was collected to measure the levels of ATP and HMGB1. As expected, both Mel and Mel@AVB obviously promoted the extracellular release of HMGB1 and ATP (fig. S11). Meanwhile, the expression of CRT, HMGB1, and HSP70 in B16F10 cells or MuM-2B cells after different treatments was detected by flow cytometry and immunofluorescence staining. As shown in Fig. 3 (D and E) and fig. S12 (A and B), it was observed that more CRT was expressed and transported from cytoplasm to cell membrane. Moreover, a large amount of HMGB1 was released from the nuclei of B16F10 cells or MuM-2B cells treated with Mel or Mel@AVB (Fig. 3, F and G, and fig. S12, C and D). In addition, increased expression of HSP70 inside B16F10 cells or MuM-2B cells in both the Mel and Mel-AVB groups was also observed (Fig. 3, H and I, and fig. S12, E and F). These results clearly demonstrated that Mel@AVB could elicit immunogenic death of cancer cells, which may further recruit different immune cells including T cells infiltrated into the tumor region, showing the potential to work synergistically with αPDL1.

### The therapeutic effect of Mel & αPDL1@AVB after vitrectomy

**Fig. 4.**
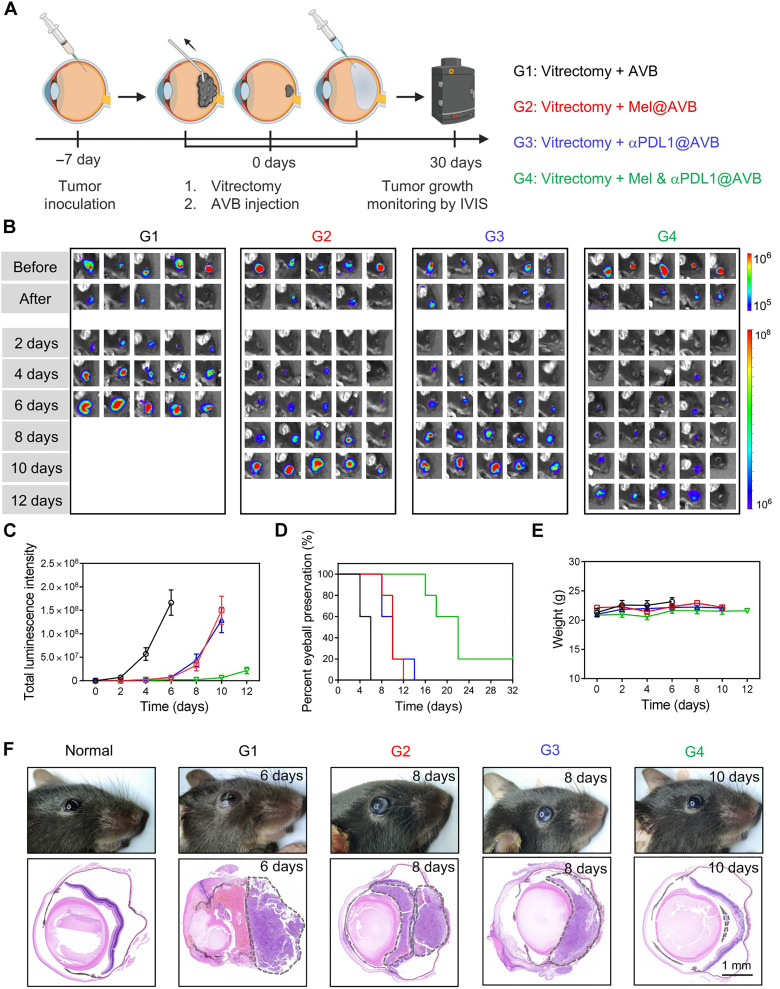
The therapeutic effect of Mel & αPDL1@AVB after vitrectomy. (**A**) Schematic shows the therapeutic procedure for choroidal melanoma. (**B**) In vivo bioluminescence images of mice bearing choroidal melanoma after different treatments (G1: Vitrectomy + AVB, G2: Vitrectomy + Mel@AVB, G3: Vitrectomy + αPDL1@AVB, and G4: Vitrectomy + Mel & αPDL1@AVB). Before, before vitrectomy; After, after vitrectomy. Five mice per group are shown. (**C**) Tumor growth kinetics corresponding to bioluminescence signals in different groups. Data are presented as means ± SEM (*n* = 5). (**D**) Percent eyeball preservation corresponding to the intensity of luminescence of mice after different treatments (*n* = 5). It was deemed necessary to remove the eyeball when the total intensity of luminescence exceeded 5 × 10^7^ p sce^−1^ cm^−2^ sr^−1^. (**E**) Weight of mice after different treatments. Data are presented as means ± SEM (*n* = 5). (**F**) Representative photographs and H&E staining images of the eyes of mice in different groups.

Inspired by the programmed release kinetics of loaded drugs in AVB and the ICD induced by Mel, we then assessed the therapeutic potential of Mel & αPDL1@AVB to inhibit the recurrence of choroidal melanoma after vitrectomy (Fig. 4A). First, the mouse choroidal melanoma model was established by injecting luciferase-expressing B16F10 cells between the retina and choroid in the right eye of each mouse. Seven days later, the mice were imaged by an in vivo fluorescence imaging system (IVIS). Strong luminescence signals (8 × 10^5^ to 1 × 10^6^ p sce^−1^ cm^−2^ sr^−1^) from cancer cells were observed, indicating the successful establishment of choroidal melanoma model (fig. S13). Meanwhile, the mice were euthanized, and their eyes were collected for dissection. As exhibited in fig. S14, obvious infiltration of cancer cells in the vitreous cavity was observed, further indicating the successful establishment of choroidal melanoma tumors. To simulate the recurrence of choroidal melanoma after vitrectomy, we removed most of the melanoma tumors. Briefly, a specially made syringe with hooked pinpoint (fig. S15) was inserted into the posterior segment via the limbus, turned gently, and drew the mixture of VB and cancer cells. The total intensity of luminescence was less than 2 × 10^5^ p sce^−1^ cm^−2^ sr^−1^ after such vitrectomy operation (Fig. 4B and fig. S13).

Next, the mice after vitrectomy were randomly divided into four groups and injected with different hydrogels into the vitreous cavity, including AVB (5 μl), Mel@AVB (5 μl, 30 μg of Mel), αPDL1@AVB (5 μl, 20 μg of αPDL1), and Mel & αPDL1@AVB (5 μl, 30 μg of Mel, 20 μg of αPDL1). Then, the growth of tumors was monitored by an in vivo fluorescence imaging system every 2 days. According to the luminescence signals from cancer cells, we found that the melanoma tumors in mice treated with blank AVB recurred 2 days after vitrectomy, and none of the mice preserved their eyeballs on day 6 (Fig. 4, B to D, and fig. S17). For Mel@AVB-treated mice and αPDL1@AVB-treated mice, although delayed recurrence of choroidal melanoma tumors was observed, their eyeball preservation period was merely extended to 12 and 14 days, respectively. Excitingly, Mel & αPDL1@AVB significantly inhibited the growth of the remaining cancer cells, and the eyeball preservation period of four of five mice was prolonged to 22 days. One of five mice in the Mel & αPDL1@AVB group were cured completely. The weights of mice in different groups during the treatment were also monitored. Nearly no weight reduction was observed in mice with different treatments, indicating that intraocular injection of Mel & αPDL1@AVB induced nearly no systemic side effects (Fig. 4E). As shown in the photos and hematoxylin and eosin (H&E) staining images of mouse eyes in different groups (Fig. 4F), the eyeball structure of mice treated with blank AVB was broken irreversibly on day 6 with full of cancer cells. The eyeballs of mice in the Mel@AVB or αPDL1@AVB group exhibited obvious leukocoria, and melanoma cancer cells occupied most of the vitreous cavity space, seriously oppressing the crystalline lens and fundus oculi on day 8. Unexpectedly, only a small fraction of melanoma cells was found in the vitreous cavity of mice treated with Mel & αPDL1@AVB on day 10. Thus, implantation of Mel & αPDL1@AVB after vitrectomy could effectively inhibit the growth of the remaining cancer cells and even cure some mice.

To explore whether this strategy could inhibit tumor metastasis, we established primary and distant tumors in both eyes of mice according to the extensively studied subcutaneous bilateral tumor model (fig. S18A). As shown in fig. S18 (B and C), when primary tumor was treated with Vitrectomy + Mel & αPDL1@AVB (30 μg of Mel, 20 μg of αPDL1), the growth of distant tumor in the left eye was also delayed. In addition, the infiltration and proliferation of antitumor CD8^+^ T cells in the distant tumor were also significantly increased (fig. S18, D and E). These results together verified that our strategy not only could control the growth of local choroidal melanoma but also has the potential to inhibit metastatic tumor growth.

To further explore the advantages of Vitrectomy + Mel & αPDL1@AVB strategy, we compared it with repetitive intravitreal injections (fig. S19A). According to the results in fig. S19 (B and C), multiple intravitreal injections at the middle stage of tumor achieved negligible therapeutic results. However, the therapeutic effect of our strategy (Vitrectomy + Mel & αPDL1@AVB), achieved significantly enhanced therapeutic effect even than that of repetitive intravitreal injections at early stage. In our treatment strategy, most of the tumor tissue was removed by vitrectomy, and then the AVB with long-term release of Mel and αPDL1 was filled to prolong the retention time of drugs and inhibit the recurrence of cancer. Thus, this Vitrectomy + Mel & αPDL1@AVB strategy was more effective in treating advanced choroidal melanoma. Multiple intravitreal injection may be more suitable for early-stage choroidal melanoma, which can achieve ideal therapeutic results and avoid greater invasiveness to the patient’s eyes. However, for middle-stage choroidal melanoma, our strategy using Vitrectomy + Mel & αPDL1@AVB can achieve better therapeutic effect and preserve the eyeball of patients.

To investigate the cytotoxicity of AVB hydrogel, retinal pigment epithelial (RPE) cells were incubated with different concentrations of AVB hydrogel for 24 hours, and the relative cell viability was measured by standard methyl thiazolyl tetrazolium (MTT) assay. As shown in fig. S20, AVB hydrogel induced negligible cytotoxicity to RPE cells. In addition, we further carried out the serum biochemistry and complete blood panel analysis 7 and 30 days after injection of AVB hydrogel (fig. S21). All the measured parameters after AVB hydrogel injection were in normal range, indicating that AVB hydrogel induced nearly no acute or chronic damage to mice. Moreover, H&E staining images of main organs collected from mice exhibited consistent histomorphological characteristics to those collected from healthy mice, which further proved that AVB hydrogel induced nearly no toxicity to normal organs of mice (fig. S22).

### Immune responses induced by Mel & αPDL1@AVB implantation

**Fig. 5.**
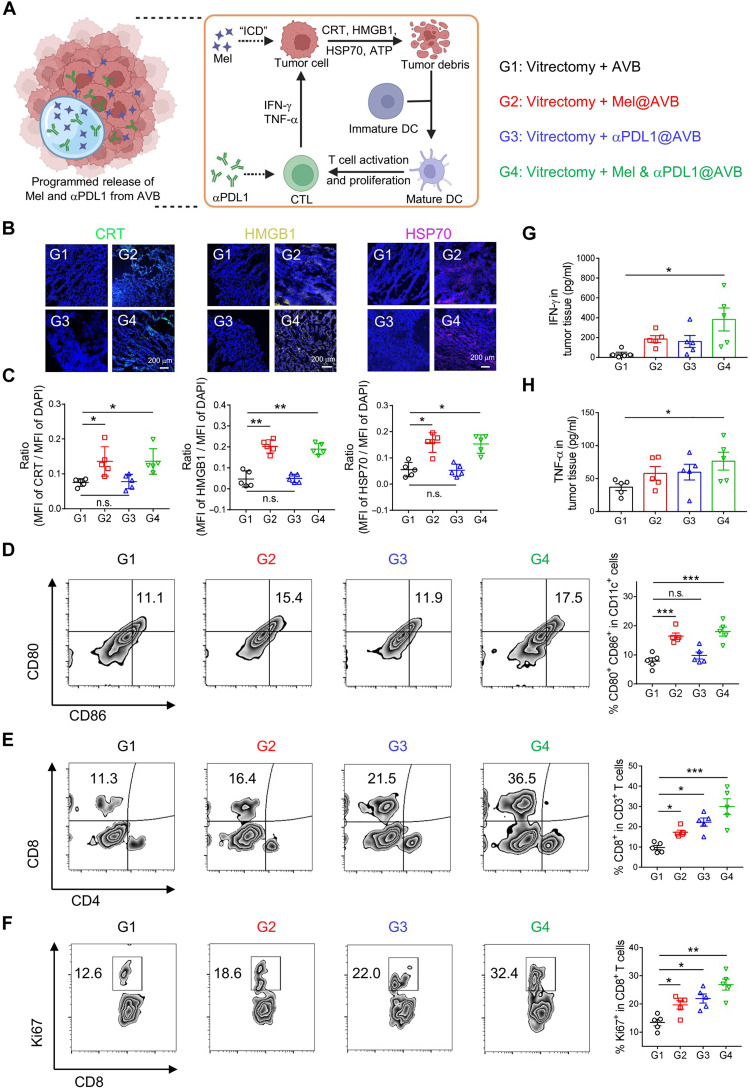
Immune responses induced by Mel & αPDL1@AVB implantation. (**A**) Schematic showing the immunologic process induced by the programmed released Mel and αPDL1 from the AVB. The early released Mel induces ICD of cancer cells and initiates a series of cytological responses, which further improves the immune response rate of subsequently released αPDL1. (**B** and **C**) Immunofluorescence staining images and statistical graphs of the expression of CRT, HMGB1, and HSP70 in choroidal melanoma with different treatments (G1: Vitrectomy + AVB, G2: Vitrectomy + Mel@AVB, G3: Vitrectomy + αPDL1@AVB, and G4: Vitrectomy + Mel & αPDL1@AVB). (**D**) Representative FACS plots and statistical graphs showing the percentage of mature DCs (CD11c^+^CD80^+^CD86^+^) in cervical lymph nodes. Data are presented as means ± SEM (*n* = 5). (**E**) Representative FACS plots and statistical graphs showing the percentage of CD8^+^ T cells (CD3^+^CD8^+^) in CD3^+^ T cells. Data are presented as means ± SEM (*n* = 5). (**F**) Representative FACS plots and statistical graphs showing the percentage of actively proliferating T cells (CD8^+^Ki67^+^) in CD8^+^ T cells. Data are presented as means ± SEM (*n* = 5). (**G** and **H**) The levels of IFN-γ and TNF-α in choroidal melanoma of mice after different treatments. Data are presented as means ± SEM (*n* = 5).

Encouraged by the excellent therapeutic effect achieved by Mel & αPDL1@AVB, we then evaluated the immune responses induced by Mel and αPDL1 in vivo (Fig. 5A). First, the eyes of mice were collected 2 days after different treatments for immunofluorescence staining. As shown in Fig. 5 (B and C), the levels of CRT, HMGB1, and HSP70 in choroidal melanoma were obviously increased in mice treated with Mel@AVB or Mel & αPDL1@AVB, demonstrating that Mel indeed could induce ICD of tumor cells in vivo. These DAMPs released during ICD could bind to pattern recognition receptors on the surface of dendritic cells (DCs) cells, initiating a series of cytological responses. Hence, the populations of DCs and T cells in choroidal melanoma were measured by flow cytometry 5 days after different treatments. As shown in Fig. 5D, the populations of mature DCs (CD11c^+^CD80^+^CD86^+^) in cervical lymph nodes in the Mel@AVB and Mel & αPDL1@AVB groups were obviously higher than those in the AVB- and αPDL1@AVB-treated groups, demonstrating that ICD induced by Mel could effectively promote the maturation of DCs in cervical lymph nodes.

In addition to DCs, we further investigated the percentages of T cells in choroidal melanoma. As we, the percentages of CD8^+^ T cells (CD3^+^CD8^+^) and actively proliferating T cells (CD8^+^Ki67^+^) were slightly increased in the Mel@AVB group, which may be attributed to the suppression of CD8^+^ T cells by programmed death pathway on tumor cells (Fig. 5, E and F). For the αPDL1@AVB group, the infiltration and proliferation of CD8^+^ T cells also showed increase, but only slightly because of insufficient antigen presentation (Fig. 5, E and F). In contrast, mice that received combination treatment using Mel & αPDL1@AVB exhibited significantly improved infiltration and proliferation of CD8^+^ T cells in their tumors (Fig. 5, E and F). Meanwhile, the levels of interferon-γ (IFN-γ) and tumor necrosis factor–α (TNF-α) in tumors of mice in the Mel & αPDL1@AVB group were found to be the highest among all tested groups (Fig. 5, G and H). Thus, Mel & αPDL1@AVB implantation could effectively trigger T cell–mediated antitumor immune responses.

### Evaluation of eyeball structure and retinal function

**Fig. 6.**
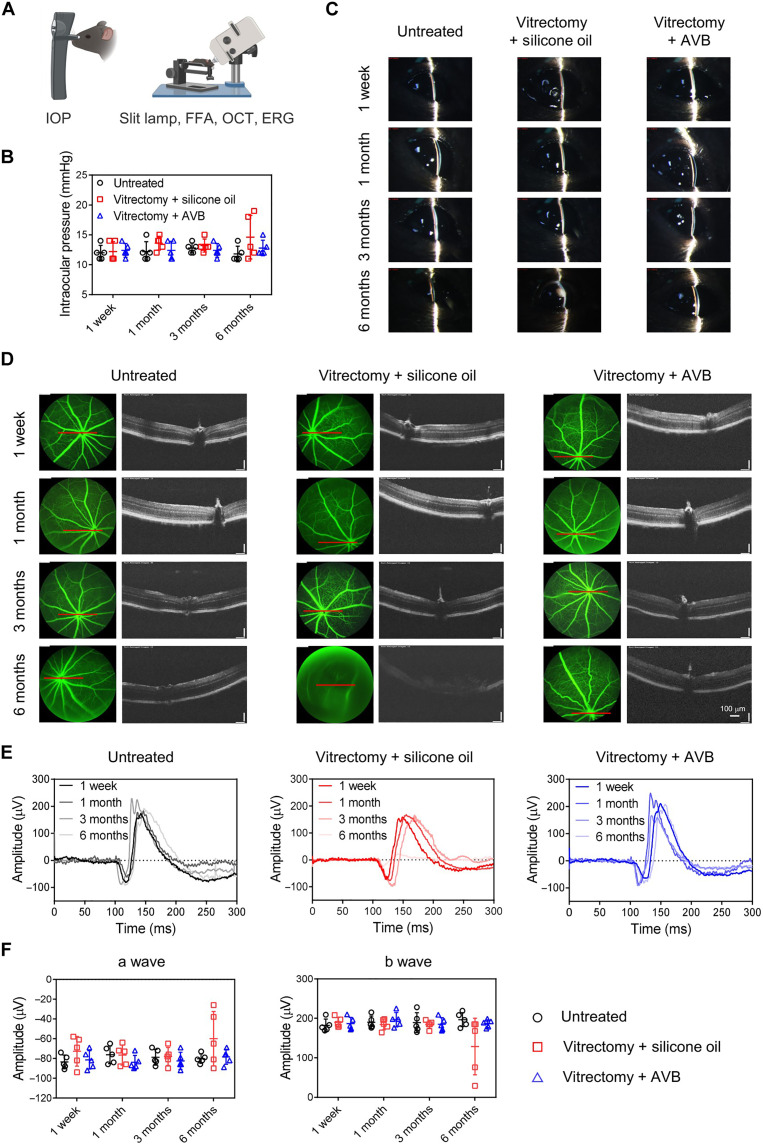
Evaluation of eyeball structure and retinal function. (**A**) Schematic showing the evaluation of eyeball structure and retinal function of mice via tonometer and retinal imaging microscope. (**B**) IOP of mice within 6 months. Data are presented as means ± SEM (*n* = 5). (**C**) Representative images of slit-lamp microscopy of the mice in different groups within 6 months. The experiments were performed five times. (**D**) Representative images of FFA (left) and OCT (right) of the mice within 6 months. The red line is the scanning position of OCT around the optic papilla. The experiments were performed five times. (**E**) Representative wave responses of ERG of mice in scotopic condition within 6 months. The experiments were performed five times. (**F**) Statistical analysis of a wave and b wave amplitudes in scotopic conditions within 6 months. Data are presented as means ± SEM (*n* = 5).

Next, we wondered whether AVB is suitable for the substitution of native VB to preserve vision after vitrectomy. To demonstrate the application of AVB after vitrectomy, healthy mice after vitrectomy were intravitreally injected with silicone oil (5 μl), a commonly used tamponade after vitrectomy in the clinic, or the AVB hydrogel (5 μl). Then, we systemically investigated the eyeball structure and retinal function of mice at different time points after surgery via a tonometer and retinal imaging microscope (Fig. 6A). As shown in Fig. 6B, the IOP of mice in AVB group was maintained between 10 and 15 mmHg, which was in the normal range, indicating that AVB did not degrade or swell in the VB to cause changes in IOP. However, two mice in the silicone oil group showed an increase in IOP at 6 months, which may be caused by the emulsification of silicone oil (*41*). In addition, we evaluated the anterior part of the eye with a slit lamp (Fig. 6C) and found that eyes injected with AVB were identical to normal eyeballs, proving that AVB induced nearly no abnormal effect on the conjunctiva and other structures in the anterior part of the eye. However, two mice in the silicone oil group developed white opaque plaques in the front of the eyes at 6 months, which could be a sign of cataracts or glaucoma (*41*). Next, retinal vascular leakage and retinal thickness were evaluated by FFA and OCT. As shown in Fig. 6D, the fundus blood vessels in the eyes of mice treated with AVB were intact without leakage. According to the OCT scanning around the optic papilla (the red-line position) (Fig. 6D and fig. S24), we found that the retinal structure and thickness of eyes in AVB group exhibited negligible differences from those in healthy mice. However, two abnormal eyes in the silicone oil group were unable to undergo normal fundus and OCT imaging at 6 months because of the influence of lesion location. Thus, comparing with silicone oil, AVB induced neatly no effect on the structure of the eye for longer filling period.

In addition to the normal structure, the evaluation of visual functions is also important for mice after injection with AVB. ERG was performed in scotopic condition to analyze the functions of photoreceptors of mice in different groups (Fig. 6, E and F, and fig. S25). As expected, the heights of the a and b waves, which are closely associated with the activity of photoreceptor cells, RPE cells, retinal bipolar cells and Müller cells (*31*), were in the normal range, indicating that our AVB exhibited nearly no effect on the visual function of mice. In contrast, the two mice in the silicone oil group with abnormal eyes showed decreased visual function (a and b waves decreased). Six months later, the mice were euthanized, and their eyes were collected for H&E staining. As shown in fig. S26, the eyes of mice treated with our AVB exhibited similar structures and morphologies compared to normal eyes. However, the fundus of the eyes in the silicone oil group showed abnormal tissue proliferation (*42*). These results collectively demonstrated that AVB should be an ideal substitution for native VB, exhibiting nearly no effects on the entire eyeball structure, maintaining the morphology and function of the eyeball for a long time, and avoiding the potential clinical complications associated with removal surgery.

## DISCUSSION

In this work, an immunotherapeutic AVB hydrogel composed of Tetra-PEG-MA and Tetra-PEG-SH was synthesized with the aim of simultaneously serving as an ideal vitreous substitute and drug reservoir to preserve vision and sustainably release immunotherapeutic drugs to inhibit the recurrence of intraocular malignant tumors. AVB hydrogels with suitable gelation time, which is matching the relevant manipulation time in the clinic, low swelling pressure, and elastic moduli comparable to those of the VB could be used as ideal vitreous-filling implants after surgical resection.

Moreover, AVB could effectively load and sequentially release chemotherapeutic drugs (Mel) and immune checkpoint inhibitors (αPDL1) with distinct release kinetics. Mel with a smaller molecular weight was released quickly from the hydrogel to induce ICD of cancer cells by releasing a large number of DAMPs to recruit various types of immune cells. Such effects working together with the subsequently released αPDL1 would be able to activate synergistic antitumor immune responses. As the result, Mel & αPDL1@AVB effectively inhibited the recurrence of the postoperative tumor and prolonged the eyeball preservation of mice bearing choroidal melanoma tumors. Meanwhile, the eyeball structure and retinal function evaluation of the implanted AVB hydrogel after 6 months indicated that the AVB should be an ideal vitreous substitute to preserve vision functions of eyes without adverse effects. Therefore, the immunotherapeutic AVB hydrogel composed of Tetra-PEG, which is the commonly used in artificial tears, has the potential to act as a crucial surgical adjunct in vitreoretinal surgery for intraocular malignant tumors in the endophytic stage of the eye. In addition, on the basis of the unique physical properties of AVB hydrogel, it has the potential to be used in a wider range of applications including retinal macular degeneration and intraocular infections.

## MATERIALS AND METHODS

### Materials, cell lines, and animals

Tetra-PEG-MA [molecular weight (MW), 10 kDa; catalog no. R-1806] and Tetra-PEG-SH (MW, 10 kDa; catalog no. R-1807) were purchased from Xi’an Ruixi Biological Technology Co Ltd. Melphalan Hydrochloride (Mel) (catalog no. M216901) was purchased from Shanghai J&K Scientific Chemical Technology Co Ltd. Anti-mouse PDL1 (catalog no. BE0101) was purchased from Bio X Cell Biotech Co Ltd. The silicone oil (Oxane 5700) was purchased from Bausch & Lomb Inc. Mouse melanoma cells (B16F10) were originally purchased from American Type Culture Collection (ATCC) and cultured in Dulbecco’s modified Eagle’s medium containing 1% penicillin-streptomycin and 10% fetal bovine serum (FBS) under standard conditions (37°C and 5% CO_2_). Human choroidal melanoma cells (MuM-2B) were also originally purchased from ATCC and cultured in RPMI 1640 medium containing 1% penicillin-streptomycin and 10% FBS under standard conditions (37°C and 5% CO_2_). The naive VB was donated by Department of Ophthalmology and Vision Science, Eye & ENT Hospital, Shanghai Medical School, Fudan University. Female C57BL/6 mice (6 to 8 weeks) and female Balb/c mice (6 to 8 weeks) were purchased from Changzhou Cavens Experimental Animal Co Ltd. All mouse experiments were conducted following the protocol approved by the Institutional Animal Care and Use Committee of Soochow University (approval no. ECSU-2019000198).

### Preparation of AVB

Appropriate quantities of Tetra-PEG-MA and Tetra-PEG-SH were separately dissolved in PBS (pH 7.4). For the MA nanogel solution, equal volumes of Tetra-PEG-MA (16 mg/ml) and Tetra-PEG-SH (4 mg/ml) were mixed. For the SH nanogel solution, equal volumes of Tetra-PEG-SH (16 mg/ml) and Tetra-PEG-MA (4 mg/ml) were mixed. Then, the two nanogel solutions were left at 4°C for 6 hours. Last, equal volumes of the two nanogel solutions were mixed to obtain AVB hydrogel with a total mass fraction of 1 wt %.

### Phase diagram

Tetra-PEG-MA and Tetra-PEG-SH were separately dissolved in PBS (pH 7.4) with the following total polymer concentration (*C*_0_) and molar proportion (*p*) of Tetra-PEG-SH. *C*_0_ = 0.8 wt %, *p* = 0.42, 0.44, 0.46, 0.48, 0.5, 0.52, 0.54, and 0.56; *C*_0_ = 1 wt %, *p* = 0.40, 0.42, 0.44, 0.46, 0.48, 0.56, 0.58, 0.60, 0.62, 0.64, and 0.66; *C*_0_ = 1.5 wt %, *p* = 0.32, 0.34, 0.36, 0.38, 0.40, 0.60, 0.62, 0.64, 0.66, 0.68, and 0.70; *C*_0_ = 2 wt %, *p* = 0.26, 0.28, 0.30, 0.32, 0.34, 0.68, 0.70, 0.72, 0.74, and 0.76; *C*_0_ = 3 wt %, *p* = 0.18, 0.20, 0.22, 0.24, 0.26, 0.76, 0.78, 0.80, 0.82, and 0.84. The corresponding set of Tetra-PEG-MA and Tetra-PEG-SH were mixed with equal volumes. After 6 hours, the gel state was determined if no flow was observed within 30 s when the vial was inverted.

### Determination of gelation time

Tetra-PEG-MA and Tetra-PEG-SH were separately dissolved in PBS (pH 7.4) with 1 wt % total polymer concentration. The mixing steps refer to the above “Preparation of AVB” section, and the gelation time was recorded using a stopwatch. It is worth noting that the sets of less than 0.6/0.4 molar proportions became hydrogels in the first step.

### Characterization of AVB

TEM images were obtained using an FEI FT20 TEM. Scanning electron microscopy images were obtained using a Zeiss Gemini 500 scanning electron microscope. The photographs were shot using a smartphone in JPEG image format. The rheological behavior was measured using a Thermo Fisher Scientific HAAKE Mars40 with P20 Ti parallel plates at 25°C. The absorbance and light transmittance were recorded using a Jasco V-750 spectrophotometer over a range of visible light (380 to 780 nm). The pH was tested by a REX PHS-25 pH meter, and the density was measured by the ethanol exclusion method. The refractive index was determined by an Abbemat 500 refractometer at 25°C. The size of nanogel was determined by a Malvern Nano-ZS90 Zetasizer. The reaction conversion was calculated by measuring the characteristic absorbance of the maleimide group in Tetra-PEG-MA at 300 nm before and after mixing up using the Jasco V-750 spectrophotometer (fig. S2, A and B). The swelling ratio was measured by weighing the AVB immersed in PBS at different time points.

The reaction conversion (proportion of Tetra-PEG-MA < 0.5) = [1 − (Tetra-PEG-MA after mixing) / (Tetra-PEG-MA before mixing)] * 100%.

The reaction conversion (proportion of Tetra-PEG-MA > 0.5) = {1 – [(Tetra-PEG-MA after mixing) – (Tetra-PEG-MA before mixing)] / (Tetra-PEG-SH before mixing)} * 100%.

The swelling ratio = (weight of AVB) / (initial weight of AVB) * 100%.

### Drug release from AVB in vitro

The drugs were loaded into AVB in the second mixing process. The drug release behavior was measured using a Transwell system (Corning, catalog no. 3422) in PBS at 37°C to mimic the physiological environment. The AVB (50 μl, 1 wt %) encapsulated with Mel (0.3 mg) and rat IgG (0.2 mg, the substitution of αPDL1) was loaded into the upper chamber, and the released Mel and IgG were collected from the bottom of the chamber at different time points, which were measured using HPLC (BioLogic DuoFlow) and the rat IgG total ELISA kit (Thermo Fisher Scientific, catalog no. 88-50490-88) separately. In addition, AVB-encapsulated RhoB- and Cy5.5-labeled rat IgG (IgG-Cy5.5) at different time points were frozen in optimal cutting temperature medium at −80°C and then cut into slices (thickness = 15 μm) using a cryotome (Lecia CM1860) at −25°C and were sealed and imaged by a confocal microscope (Zeiss LSM 800).

### Retention of drug/antibody in vivo

RhoB- and Cy5.5-labeled rat IgG (IgG-Cy5.5) were used as substitutes for Mel and αPDL1, respectively, for fluorescence imaging. Five microliters of mixture containing RhoB (30 μg) and IgG-Cy5.5 (20 μg) or 5 μl of AVB (1 wt %) encapsulating RhoB (30 μg) and IgG-Cy5.5 (20 μg) were injected separately into the right eyeballs of Balb/c mice using intravitreal injection with a microinjection needle (Hamilton, catalog no. 7653-01). The mice were then monitored by an in vivo fluorescence imaging system (PerkinElmer IVIS Lumina III) at different time points.

### Evaluation of CRT, HMGB1, HSP70, and ATP

Immunofluorescence staining of CRT, HMGB1, and HSP70 was performed. For the in vitro experiment, B16F10 cells or MuM-2B cells cultured in a 24-well plate were treated with AVB (10 μl), Mel (80 μg in 1 ml of medium), or Mel@AVB (80 μg of Mel in 10 μl of AVB) for 6 hours, fixed with 0.25% paraformaldehyde, and blocked with FBS. Next, the cells were stained with anti-CRT (Abcam, catalog no. ab92516), anti-HMGB1 (Abcam, catalog no. ab79823), and anti-HSP70 (Abcam, catalog no. ab181606) separately overnight at 4°C with a dilution of 1:1000 (0.1% Triton X-100 was used for cell membrane perforation before staining with anti-HMGB1). Then, the slides were washed with PBS and incubated with fluorescence-labeled secondary goat anti-rabbit IgG H&L (Alexa Fluor 488) (1:400) (Abcam, catalog no. ab150077) at room temperature for 1 hour, and the nuclei were stained with 4′,6-diamidino-2-phenylindole for 10 min. Last, the cells were sealed and imaged by a confocal microscope (Zeiss LSM 800). For the in vivo experiment, the mice were euthanized on day 2, and their tumors were collected and frozen in optimal cutting temperature medium at −80°C. After 6 hours, the tumors were cut into slices (thickness = 10 μm) using a cryotome (Lecia CM1860) at −25°C, mounted on slides, and fixed with 4% paraformaldehyde. Then, the same staining procedure was performed as in the cell experiment.

For flow cytometry analysis, B16F10 cells or MuM-2B cells cultured in a 12-well plate were treated with AVB (20 μl), Mel (160 μg), or Mel@AVB (160 μg of Mel in 20 μl of AVB) for 6 hours (treated for 12 hours in terms of HMGB1). Then, the cells were collected in Eppendorf tubes, fixed with 0.25% paraformaldehyde, and blocked with anti-CD16/32 (BioLegend, 101302) antibodies. Next, these cells were stained with anti-CRT, anti-HMGB1, and anti-HSP70 separately for 0.5 hours at room temperature at a dilution of 1:500 (0.1% Triton X-100 was used for cell membrane perforation before staining with anti-HMGB1). Then, the cells were washed with PBS and incubated with fluorescence-labeled secondary goat anti-rabbit IgG H&L (Alexa Fluor 488) (1:800) at room temperature for 0.5 hours. Last, cells for each sample were tested using a BD AccuriC6 plus flow cytometer and analyzed by FlowJo software (Ver. 10.0.7).

For determination of released HMGB1 and ATP, the culture medium of B16F10 cells after different treatments was collected and measured by an ELISA kit (Beyotime, catalog no. PH406) and an ATP assay kit (Beyotime, catalog no. S0027).

### Vitrectomy in a mouse model

The anesthetized mice were fastened to the platform of a stereomicroscope, and then a 30-G needle was inserted with the best position, angle, and depth to ensure no effect on the normal tissue under the microscope and the guidance of ophthalmologists. Next, a specially made syringe with the hooked pinpoint (fig. S15) was inserted into the posterior segment via the pore to gently draw the mixture of VB and tumor. Last, the just mixed solution of equal amounts of MA nanogel and SH nanogel was injected into the posterior segment via the pore using a microinjection needle, followed by a drop of the antibiotic eyedrop. The next day, IOPs were measured to ensure that AVB reached equilibrium in the vitreous cavity.

### In vivo treatment of choroidal melanoma

B16F10 or B16LS9 are two cell lines derived from murine cutaneous melanoma tissues and have been widely used to establish choroidal melanoma in previous studies (*43*–*45*). To establish the choroidal melanoma mouse model, 5 × 10^5^ luciferase B16F10 cells were injected into the middle between the retina and choroid in the right eye of each mouse. Seven days later, the tumor cells had invaded the vitreous cavity (fig. S14). Then, the mice were randomly divided into four groups (five mice per group), and vitrectomy was performed to remove the tumor until the total intensity of luminescence was less than 2 × 10^5^ p sce^−1^ cm^−2^ sr^−1^ (fig. S13). Then, 5 μl of AVB, Mel@AVB (30 μg of Mel), αPDL1@AVB (20 μg of αPDL1), or Mel & αPDL1@AVB (30 μg of Mel and 20 μg of αPDL1) were injected separately into the vitreous cavity. Last, tumors inside the eyes were monitored by an in vivo fluorescence imaging system (PerkinElmer IVIS Lumina III) every 2 days. It was deemed necessary to remove the eyeball when the total intensity of luminescence exceeded 5 × 10^7^ p sce^−1^ cm^−2^ sr^−1^ because the melanoma cancer cells occupied most of the vitreous cavity space and increased the risk of metastasis (fig. S16).

To establish the metastatic tumor model, 4 days after inoculation of the primary tumor in the right eye of each mouse, an equal amount of tumor cells was injected into the left eye to establish a distant tumor (fig. S18), and the primary tumor was performed with Vitrectomy + Mel & αPDL1@AVB (30 μg of Mel and 20 μg of αPDL1) on day 7. The tumors inside the eyes were monitored by an in vivo fluorescence imaging system (PerkinElmer IVIS Lumina III) every 2 days.

To explore the therapeutic efficacy repetitive intravitreal injections (fig. S19), mice bearing choroidal melanoma were divided into three groups: G1, untreated; G2, intravitreal injection of 30 μg of Mel and 20 μg of αPDL1 for three times at −4, −2, and 0 days (at early stage); and G3, intravitreal injection of 30 μg of Mel and 20 μg of αPDL1 for three times at 0, 2, and 4 days (at middle stage) (the total intravitreal injection dose is three times that of Mel & αPDL1@AVB). The tumors inside the eyes were monitored by an in vivo fluorescence imaging system (PerkinElmer IVIS Lumina III) every 2 days.

### H&E staining

Eyeballs were collected and fixed in eyeball-fixed liquid, dehydrated with ethanol, embedded in paraffin, and cut into sections (8 μm). Then, the tissue sections were stained with H&E and imaged using an optical microscope (DM 4000).

### Flow cytometry

Tumors and cervical lymph nodes harvested from mice were collected and homogenized into the single-cell suspensions. Then, the cells were blocked with anti-CD16/32 (BioLegend, catalog no. 101302) antibodies to avoid nonspecific adsorption and stained with the following antibodies according to the manufacturer’s instructions: fluorescein isothiocyanate (FITC)–anti-CD45 (BioLegend, catalog no. 147710), Peridinin-Chlorophyll-Protein Complex (PerCP)-CD11c (BioLegend, catalog no. 117326), allophycocyanin (APC)–anti-CD80 (BioLegend, catalog no. 104714), phycoerythrin (PE)–anti-CD86 (BioLegend, catalog no. 105106), PerCP–anti-CD3 (BioLegend, catalog no. 100326), APC–anti-CD4 (BioLegend, catalog no. 100412), PE–anti-CD8 (BioLegend, catalog no. 100708), and FITC–anti-Ki67 (BioLegend, catalog no. 151212). A total of 1.0 × 10^5^ events in the ungated flow chart in each sample were collected using a C6 plus flow cytometer and analyzed by FlowJo software (Ver. 10.0.7). The gating strategy is given in fig. S23.

### IFN-γ and TNF-α detection in tumor tissue

The collected tumors were ground fully in radioimmunoprecipitation assay lysis buffer at 4°C and centrifuged to remove the precipitates three times. The levels of IFN-γ and TNF-α were measured by mouse ELISA kits (eBioscience, 88-7314-88 and 88-7324-88) according to the manufacturer’s instructions.

### Evaluation of eyeball function

IOP was measured by instantaneous contact with the center of the cornea using the Icare Tonolab tonometer. Slit-lamp microscopy, FFA, OCT, and ERG were performed using a Micron IV retinal imaging microscope (Phoenix Research Laboratories Inc.) equipped with the corresponding modules. Note that the mice were dark adapted in advance for 12 hours for ERG examination, and a single-flash stimulus at an intensity of 5 log(cd/m^2^) for a duration of 5 ms was used to elicit the rod and mixed cone/rod responses.

### Statistical analysis

Statistical analysis was performed via GraphPad Prism software 7. All data are presented as means ± SEM. Two-tailed Student’s *t* test was used for two-group comparisons, and one-way analysis of variance (ANOVA) with a Tukey post hoc test was used for multiple comparisons. Survival benefit was assessed using a log-rank test. The threshold for statistical significance was *P < 0.05; **P < 0.01; ***P < 0.001. All figure illustrations were created with BioRender.com.
