# Supplementary material for: An immunotherapeutic artificial vitreous body hydrogel to control choroidal melanoma and preserve vision after vitrectomy
Source: Sci Adv. 2023 Nov 1;9(44):eadh1582. doi: 10.1126/sciadv.adh1582 (PMC10619924; doi:10.1126/sciadv.adh1582)
Supplement: Supplementary file 1 — Figs. S1 to S26 [file sciadv.adh1582_sm.pdf]

Science Advances
AAAS

Supplementary Materials for

An immunotherapeutic artificial vitreous body hydrogel to control choroidal melanoma and preserve vision after vitrectomy

Muchao Chen *et al.*

Corresponding author: Qian Chen, chenqian@suda.edu.cn

Sci. Adv. **9**, eadh1582 (2023)
DOI: 10.1126/sciadv.adh1582

This PDF file includes:

Figs. S1 to S26

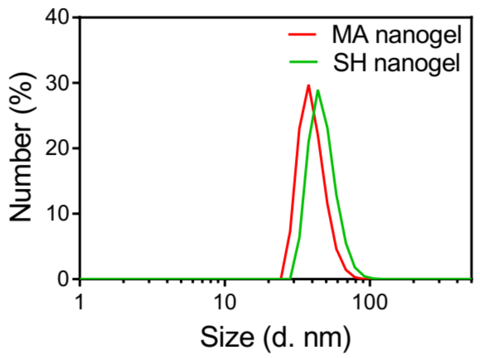

figure S1: The hydrodynamic diameter of MA nanogel and SH nanogel.

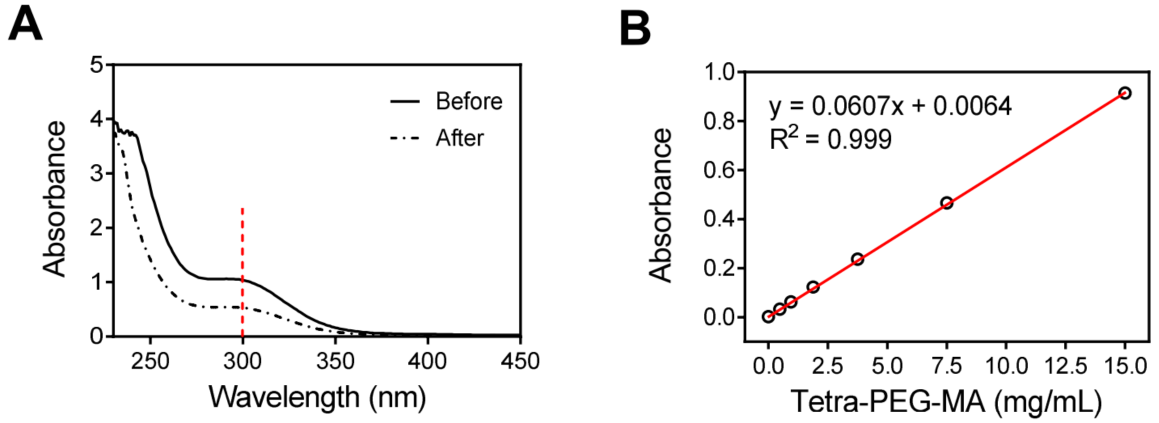

figure S2: (A) The UV-Vis absorbance of Tetra-PEG-MA before and after being mixed with Tetra-PEG-SH. **(B)** The concentration-dependent absorbance curve of Tetra-PEG-MA at 300 nm.

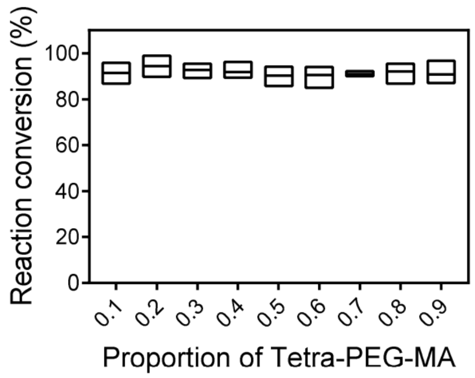

figure S3: The reaction conversion of Tetra-PEG-MA mixed with Tetra-PEG-SH in different proportions.

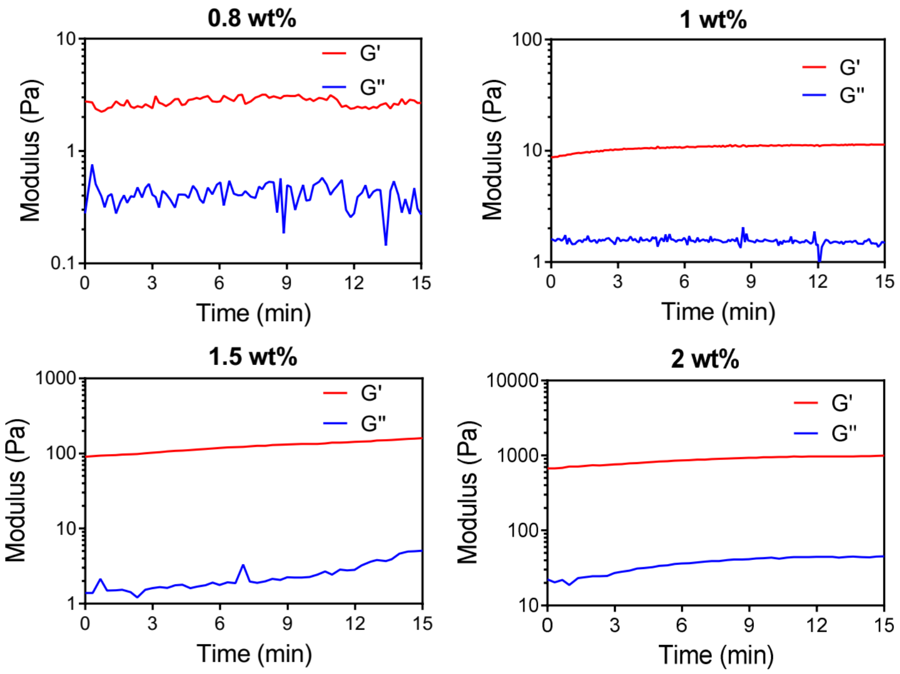

figure S4: The rheological behavior of AVB with different mass fraction. G': storage modulus; G'': loss modulus.

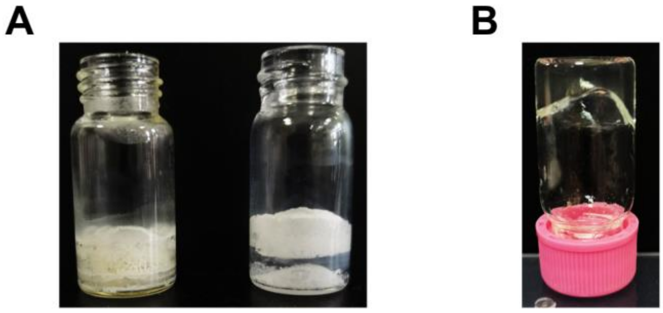

figure S5: (A) Photograph of lyophilized powders of two nanogels (Left: SH nanogel; Right: MA nanogel). **(B)** Photograph of AVB hydrogel formed from the two nanogel lyophilized powder.

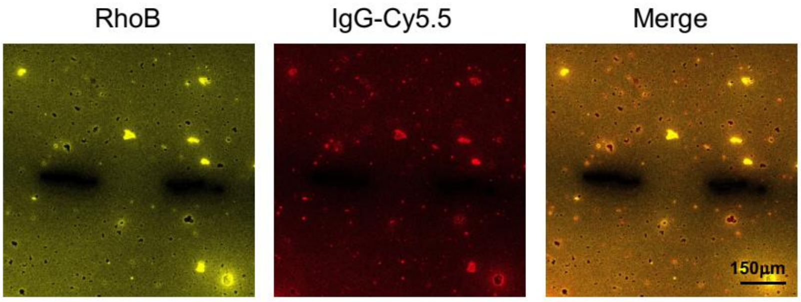

figure S6: The fluorescence of encapsulated RhoB and IgG-Cy5.5 in the AVB hydrogel.

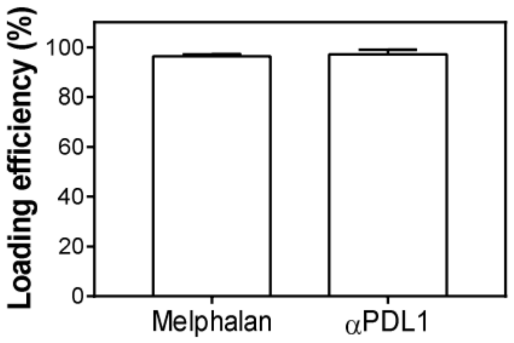

figure S7: The loading efficiency of Mel and αPDL1 in the AVB hydrogel.

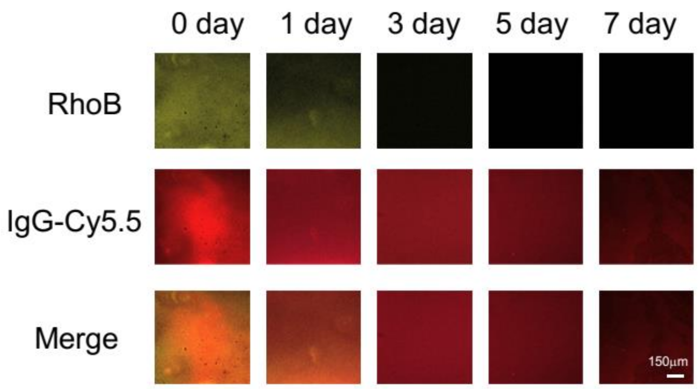

figure S8: The release behaviors of encapsulated RhoB and IgG-Cy5.5 visualized by fluorescence in the AVB hydrogel at different time points.

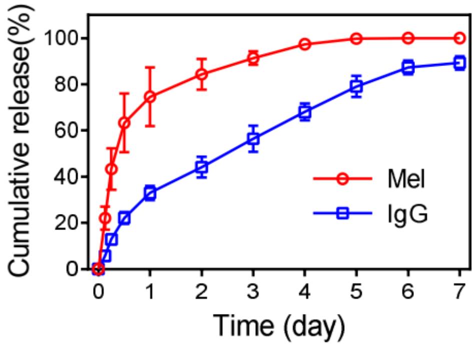

figure S9: The quantitative release profiles of Mel and IgG from AVB. Data are presented as mean ± s.e.m (n = 3)

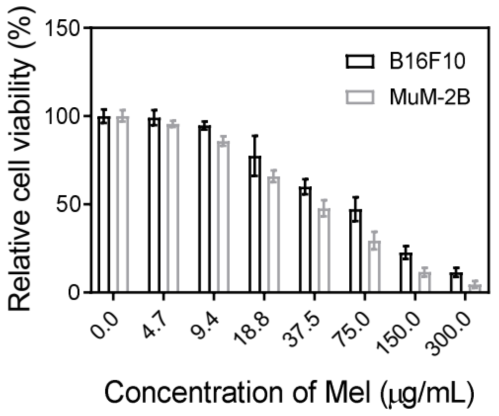

figure S10: Relative B16F10 and MuM-2B cell viability of AVB containing a range of concentrations of Mel after incubation with cells for 24 hours. Data are presented as the mean ± s.e.m. (n = 6)

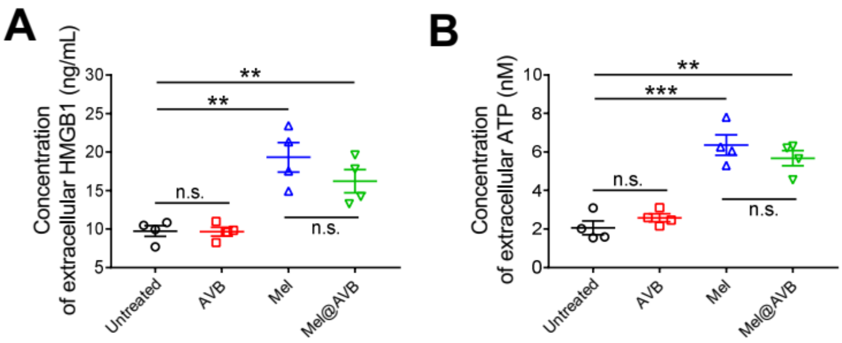

figure S11: (A) & (B) The level of HMGB1 and ATP in culture medium after different treatments. Data are presented as mean ± s.e.m (n = 4)

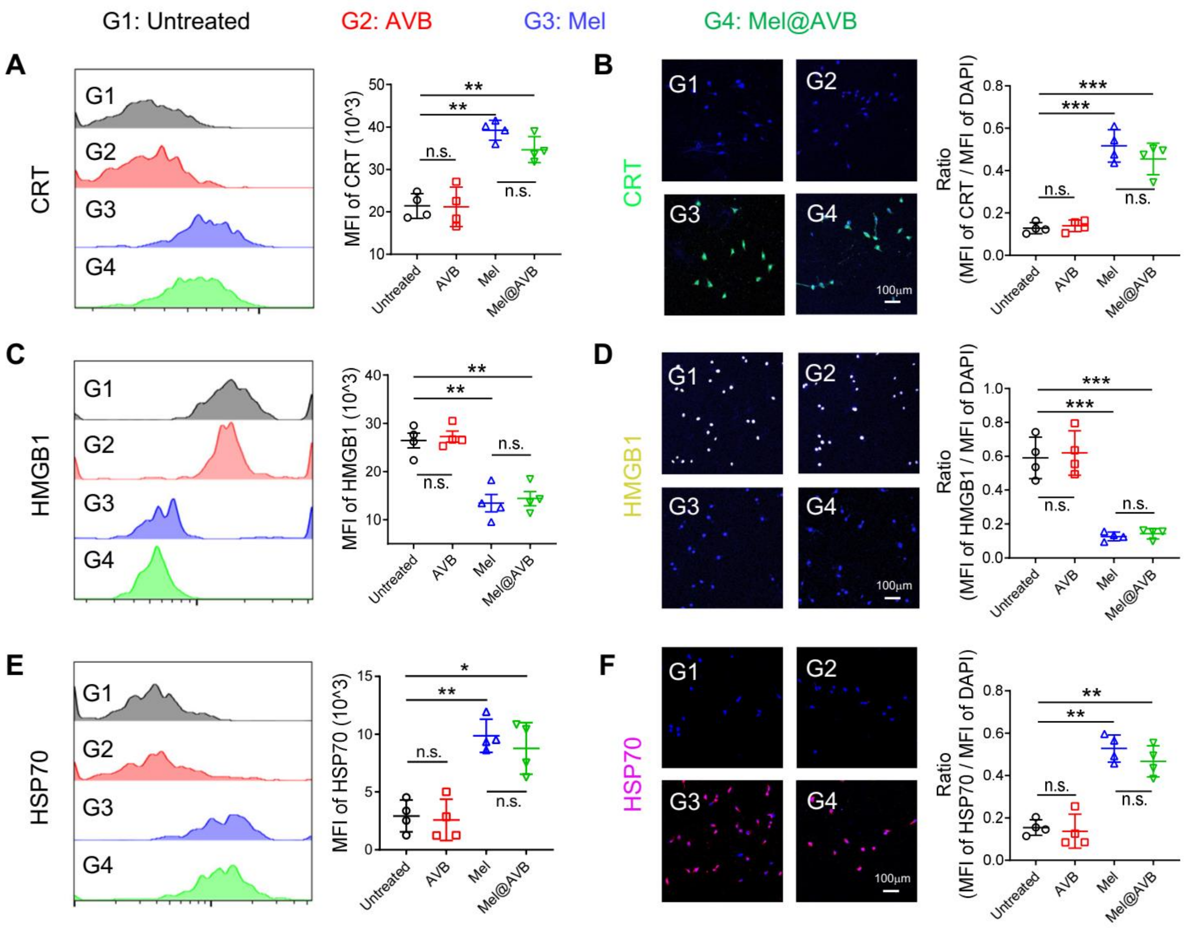

figure S12: (A), (C) & (E) Representative FACS histograms and statistical graphs of CRT, HMGB1 and HSP70 expression on MuM-2B tumor cells with different treatments (G1: Untreated, G2: AVB, G3: Mel, G4: Mel@AVB). Data are presented as the mean ± s.e.m. (n = 4). **(B), (D) & (F)** Immunofluorescence staining images and statistical graphs of CRT, HMGB1 and HSP70 expression on MuM-2B tumor cells with different treatments. Data are presented as the mean ± s.e.m. (n = 4).

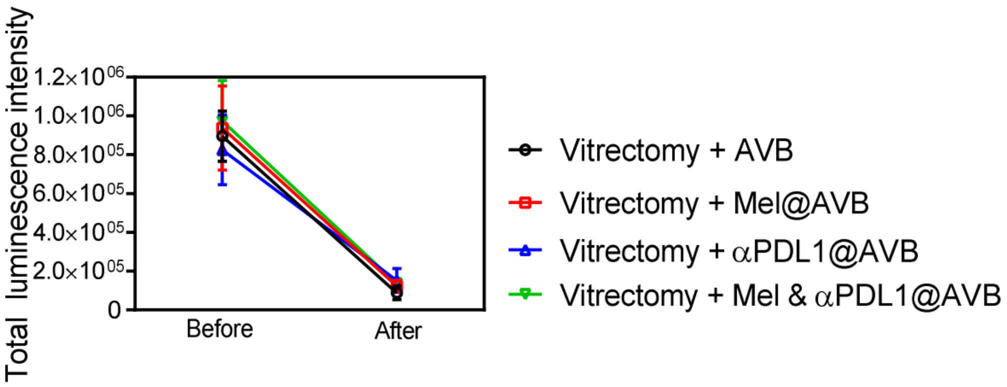

figure S13: The intensity of luminescence of choroidal melanoma before and after vitrectomy.

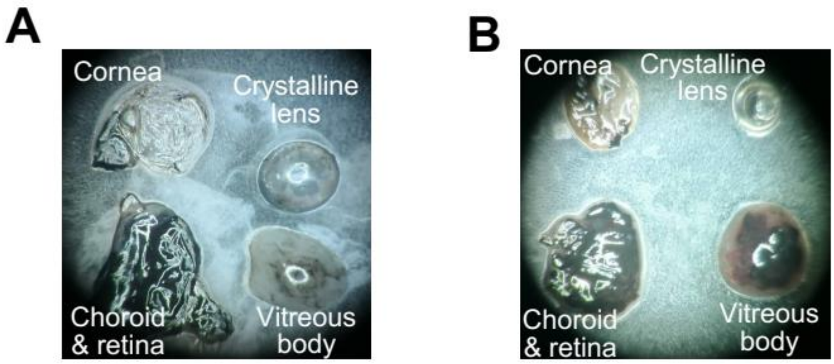

figure S14: (A) & (B) Anatomical photographs of different tissues of the eyeball. (Left: normal eyeball. Right: choroidal melanoma model.)

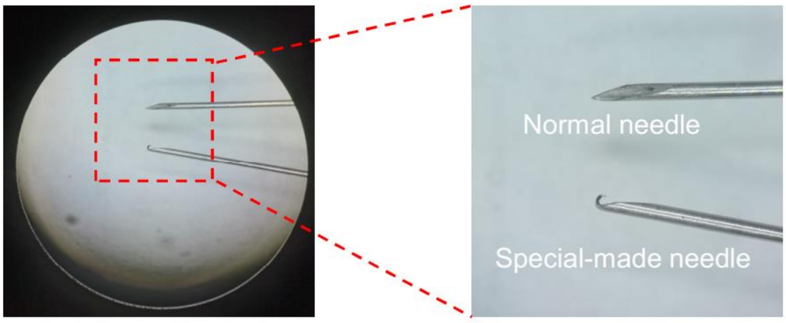

figure S15: The photograph of the special-made needle used for vitrectomy.

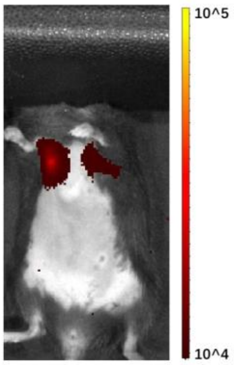

figure S16: Representative in vivo bioluminescence images of mice with choroidal melanoma metastases.

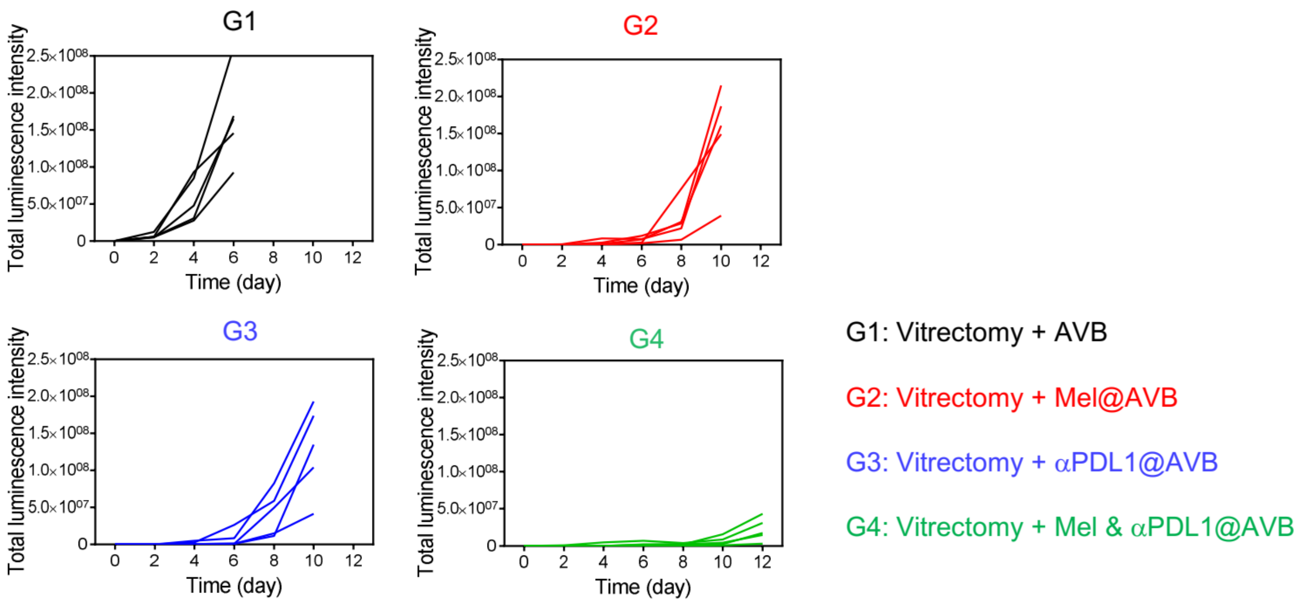

figure S17: Tumor growth kinetics of each group (n = 5).

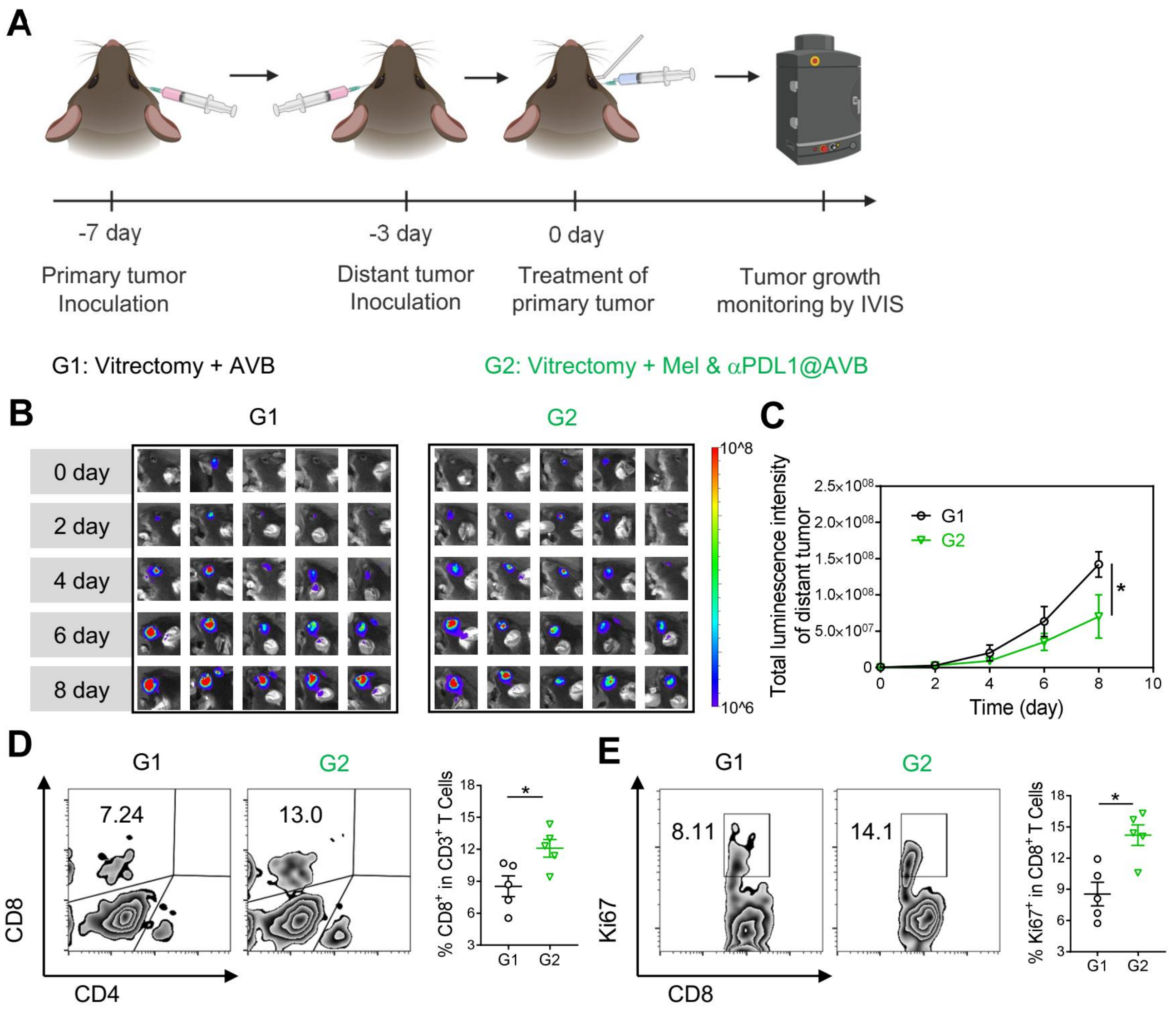

figure S18: (A) Schema showing the therapeutic procedure for metastatic choroidal melanoma. (Right: primary tumor, Left: distant tumor) **(B)** In vivo bioluminescence images of mice bearing distant choroidal melanoma after different treatments. Five mice per group are shown. **(C)** Tumor growth kinetics corresponding to bioluminescence signals of distant choroidal melanoma in different groups. **(D)** Representative FACS plots and statistical graphs showing the percentage of $CD8^+$ T cells ($CD3^+CD8^+$) in $CD3^+$ T cells. **(E)** Representative FACS plots and statistical graphs showing the percentage of proliferating T cells ($CD8^+Ki67^+$) in $CD8^+$ T cells. Data are presented as the mean ± s.e.m. (n = 5).

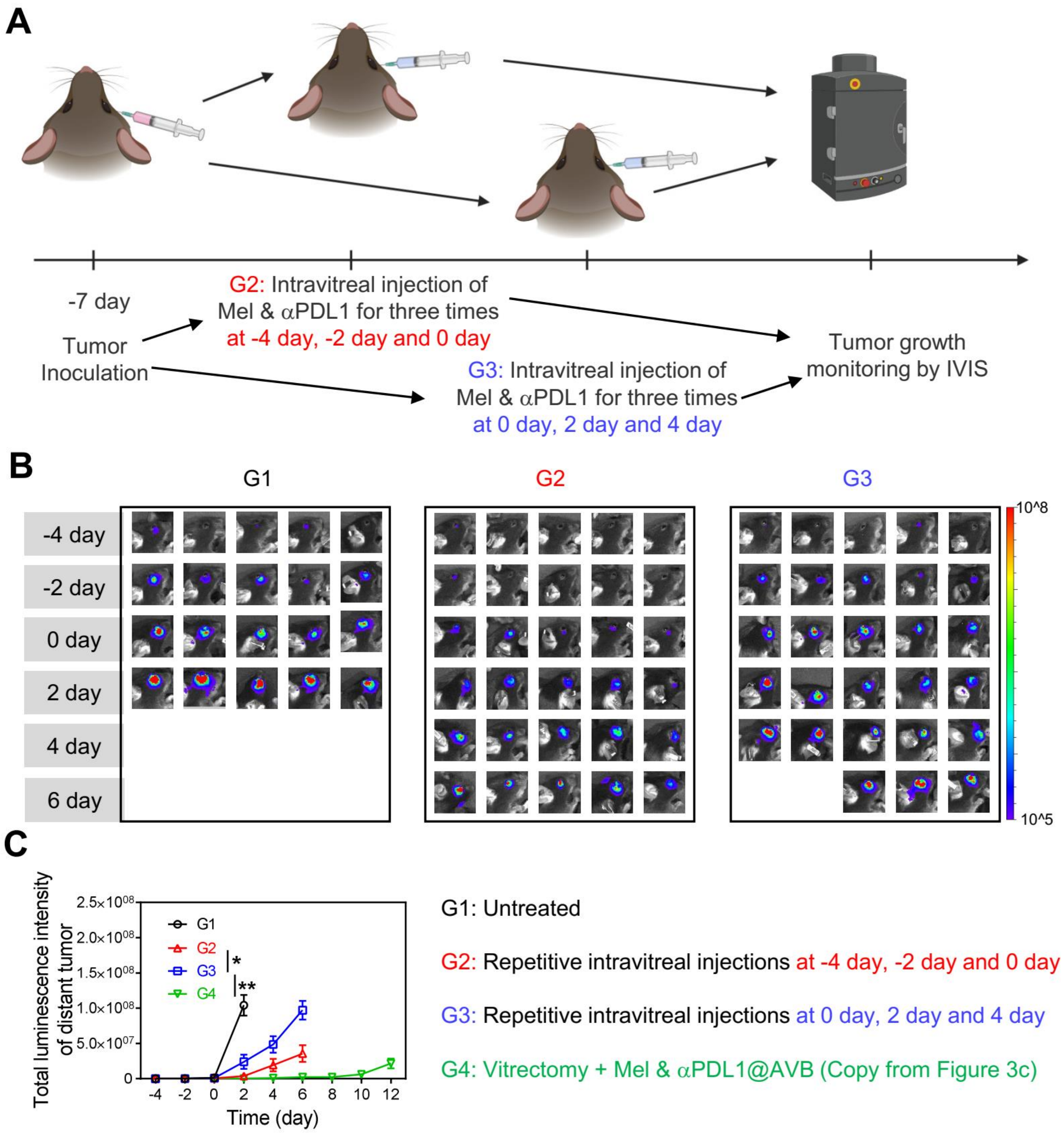

figure S19: (A) Schema showing the therapeutic procedure of multiple intravitreal injections of Mel & αPDL1 for choroidal melanoma at early and middle stage. **(B)** In vivo bioluminescence images of mice bearing choroidal melanoma in different groups. Five mice per group are shown. **(C)** Tumor growth kinetics corresponding to bioluminescence signals of choroidal melanoma in different groups. Data are presented as the mean ± s.e.m. (n = 5).

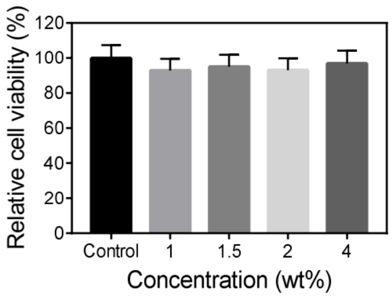

figure S20: Relative viabilities of RPE cells after incubated with different concentrations of AVB hydrogel for 24 h. Data are presented as the mean ± s.e.m. (n = 6).

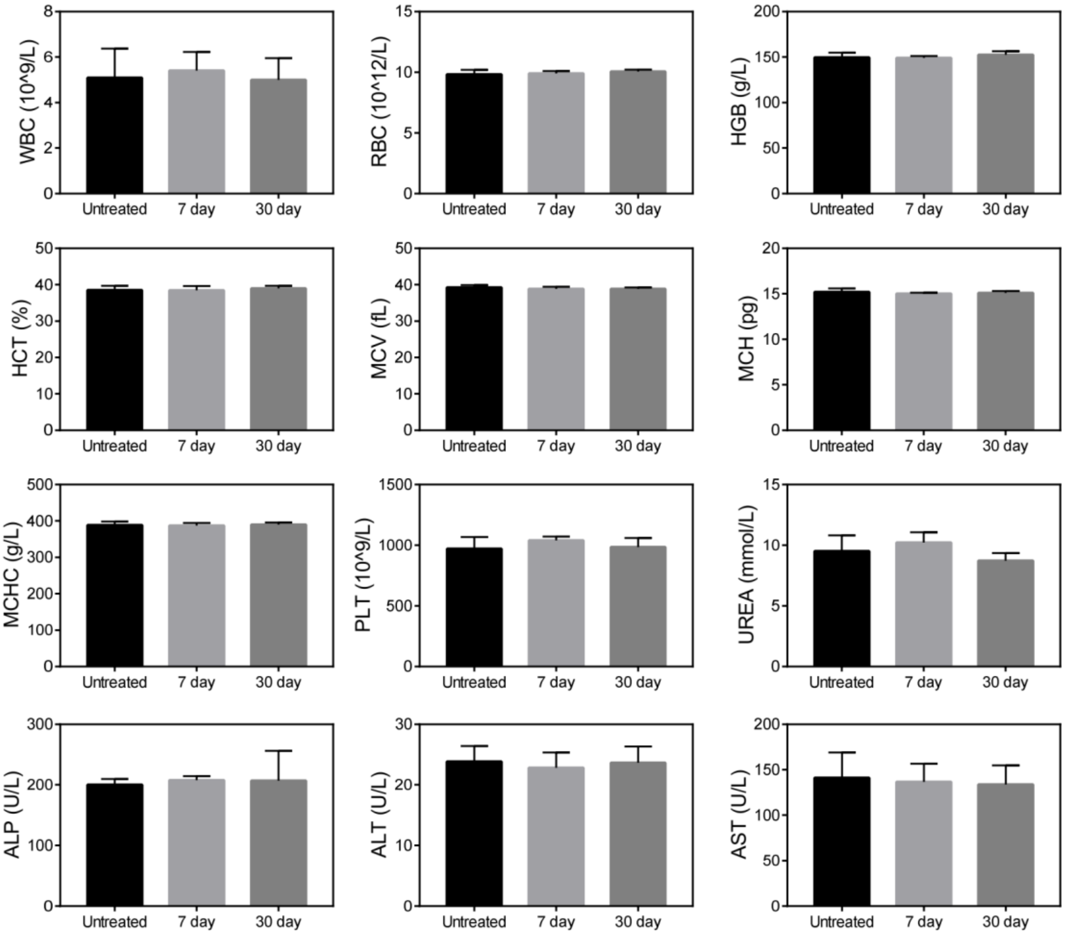

figure S21: The serum biochemistry and complete blood panel analysis of mice after injection of Mel&PDL1@AVB on day 7 and day 30. WBC: white blood cells, RBC: red blood cells, HGB: hemoglobin, HCT: hematocrit, MCV: mean corpuscular volume, MCH: mean corpuscular hemoglobin, MCHC: mean corpuscular hemoglobin concentration, PLT: platelets, UREA: Urea, ALP: alkaline phosphatase, ALT: alanine aminotransferase, AST: aspartate aminotransferase. Data are presented as the mean ± s.e.m. (n = 5)

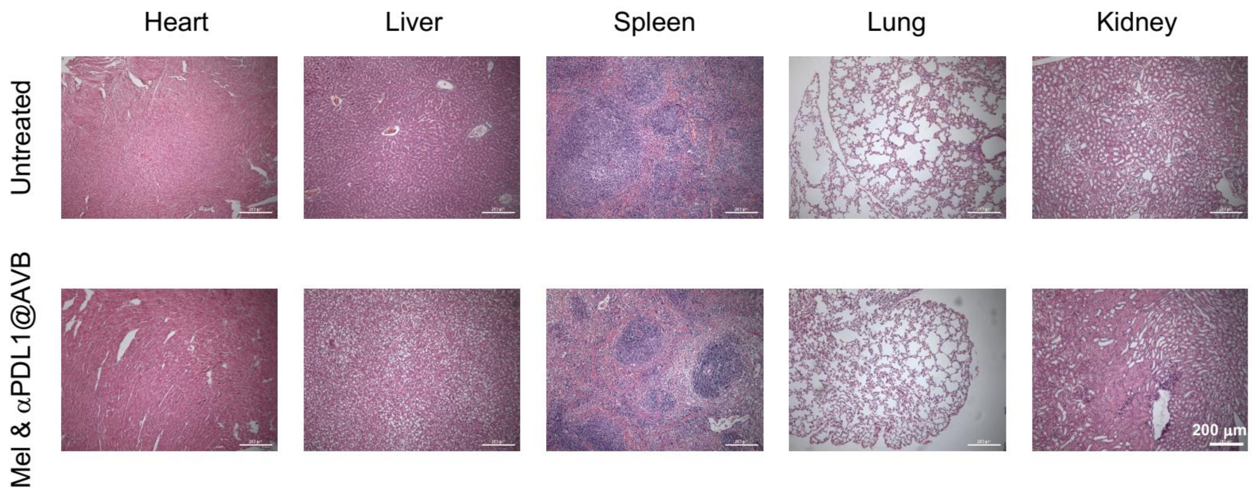

figure S22: Hematoxylin eosin (H&E) staining images of main organs (heart, liver, spleen, lung, and kidney) collected from mice in different groups on day 30.

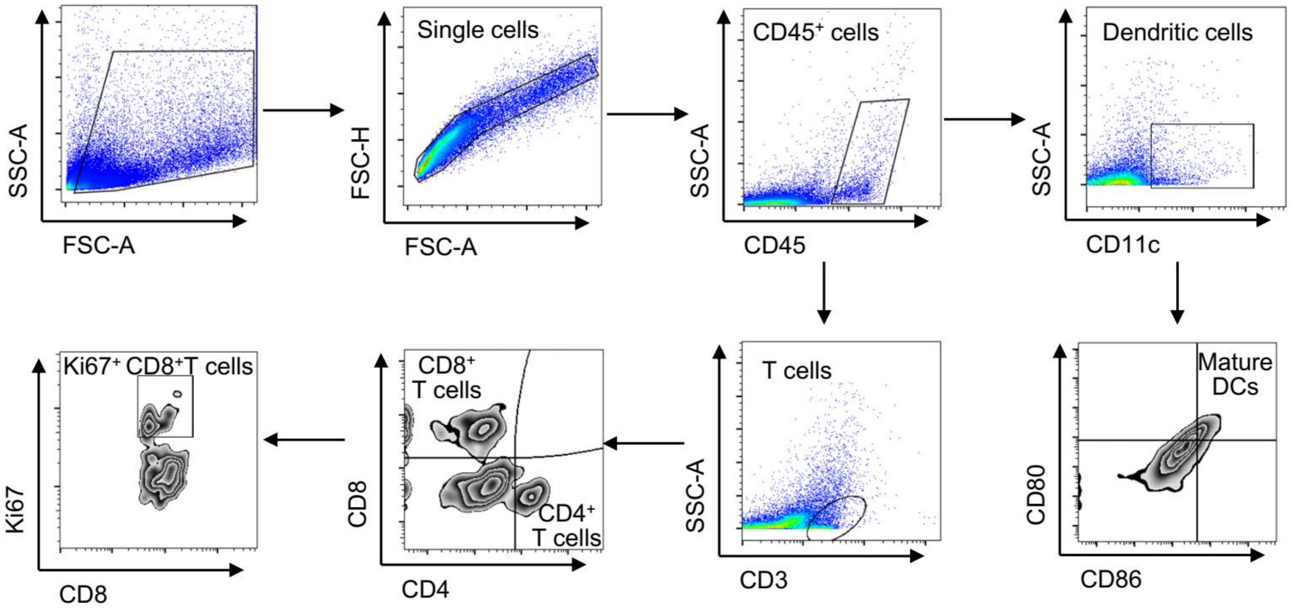

figure S23: The gating strategy of flow cytometry.

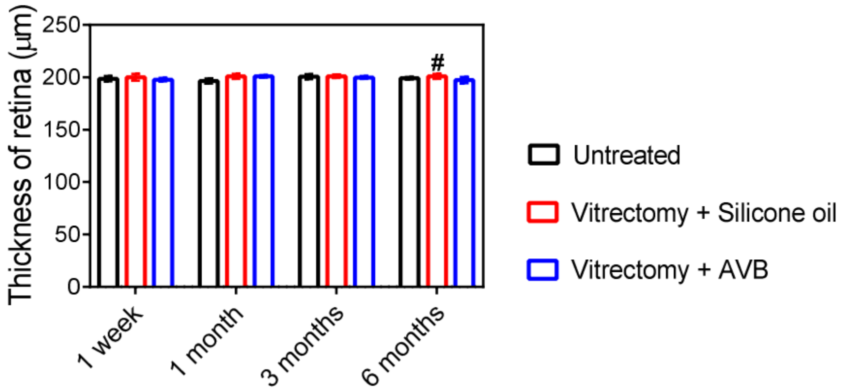

figure S24: Statistic thickness of retina in the OCT images. Data are presented as mean ± s.e.m. (n = 5). # group represented statistics from three mice with normal eyes because OCT imaging was not available for abnormal mouse eyes

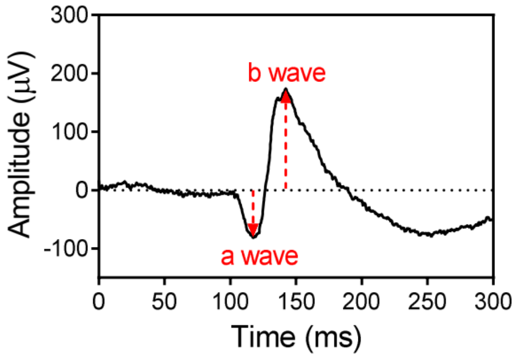

figure S25: The diagram of quantification of a wave and b wave.

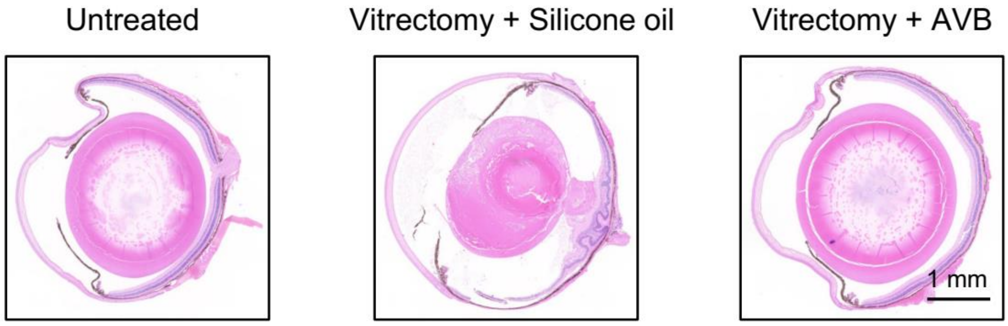

figure S26: The representative H&E staining images of eyes of mice in different groups.
